# MicroRNAs: Novel Targets in Hepatic Ischemia–Reperfusion Injury

**DOI:** 10.3390/biomedicines10040791

**Published:** 2022-03-29

**Authors:** Holly Ingram, Murat Dogan, James D. Eason, Cem Kuscu, Canan Kuscu

**Affiliations:** Transplant Research Institute, James D. Eason Transplant Institute, Department of Surgery, School of Medicine, University of Tennessee Health Science Center, Memphis, TN 38163, USA; hingram5@uthsc.edu (H.I.); mdogan@uthsc.edu (M.D.); jeason1@uthsc.edu (J.D.E.); ckuscu1@uthsc.edu (C.K.)

**Keywords:** microRNA, ischemia–reperfusion injury (IRI), liver transplantation, biomarkers, inflammation

## Abstract

Hepatic ischemia–reperfusion injury (IRI) is one of the main factors for early allograft dysfunction (EAD), which may lead to graft rejection, graft loss, or shortened graft life in liver transplantation. Hepatic IRI appears to be inevitable during the majority of liver procurement and transportation of donor organs, resulting in a cascade of biological changes. The activation of signaling pathways during IRI results in the up- and downregulation of genes and microRNAs (miRNAs). miRNAs are ~21 nucleotides in length and well-characterized for their role in gene regulations; they have recently been used for therapeutic approaches in addition to their role as biomarkers for many diseases. miRNAs that are associated with hepatic IRI in in vitro and in vivo animal models are comprehensively summarized in this review. In those studies, the manipulation of miRNAs has been shown for the inhibition of aggravated immune response, reduction of apoptosis, stimulation of tissue repair, and enhancement of cell recovery to attenuate liver damage. Therefore, the utilization of liver-specific miRNA holds great potential as a therapeutic agent to improve early allograft dysfunction, hepatic injury, and patient outcome.

## 1. Introduction

Liver transplantation is the only curative treatment for end-stage liver diseases. Despite the improvements in immunosuppression, graft loss after liver transplantation is still a major obstacle. One of the main problems is early allograft dysfunction (EAD) due to insult in the organ during transportation along with donor characteristics. One of the major risk factors for EAD is hepatic ischemia–reperfusion injury (IRI) [1]. IRI is a life-threatening condition which is caused by ischemia occurring during transportation of liver from donor site to recipient site and restoring the blood flow to the recipient following transplantation. Hepatic IRI progression has complex pathophysiology which activates multiple signaling pathways, including ischemia-induced cell damage and reperfusion-induced inflammation. If this progressive liver damage stays irreversible, it can lead to multi-organ dysfunction syndrome (MODS) or systemic inflammatory response syndrome (SIRS), leading to mortality in transplant patients [1,2].

IRI exacerbates cellular dysfunction due to the additional liver damage sustained during blood restoration to ischemic tissue [3]. Reactive oxygen species (ROS) mediate reperfusion through endothelial dysfunction and an inflammatory response, resulting in tissue damage [1,4,5]. Additional factors of IRI include oxidative stress, inflammatory cascades, cytokine storms, and Kupffer cells (KCs)/neutrophil activation [6,7].

Hypoxia of the tissues during transplantation results in decreased ATP from electron transport chain dysfunction. Due to the inhibition of ATP production, glucose metabolism results in an increase in lactic acid, which decreases the tissue pH [1,4]. Further metabolic dysregulation leads to a loss of ionic gradients from the inability of ATP-dependent ionic pumps to regulate Na^+^, K^+^, and Ca^2+^ [5]. ATP is degraded into hypoxanthine during ischemic conditions. When the tissue is reperfused, xanthine oxidase is catalyzed by the sudden increase in oxygen, resulting in the degradation of hypoxanthine to uric acid and superoxide anion [8]. Then, the highly reactive superoxide can be converted into hydrogen peroxide and the hydroxyl radical, which cause an inflammatory response through the dysregulation of cell permeability [8]. ROS continues to activate endothelial cells through NF-kB activation. The elevation of NF-kB causes systemic inflammation through the increased expression of cytokines such as TNF-a, IL-1, IL-6, and IL-8 [7,8]. Therapeutic implementations are necessary to reduce the inflammatory cascade which can result in organ failure and ultimately death.

IRI is one of the central causes of disease and death through stroke, myocardial infarction, and organ transplantations [1,6,7]. The identification of new therapeutic approaches to treat hepatic IRI is vital to improve graft function and survival considering the current national shortage of donor organs [1]. Tissue damage is increased through feedback mechanisms of IRI, which could potentially be intervened in by targeting inflammatory pathways [3]. The mechanisms of IRI pathogenesis are highly integrated, but the discovery of microRNAs (miRNAs) has shown great promise in improving graft survival after liver transplantation when used as biomarkers for diagnosis [9,10]. The inhibition or upregulation of miRNAs during IRI specifically, dependent on their role within the pathophysiology of the disease, could ameliorate liver injury [3]. In this review, we will summarize the role of different miRNAs in hepatic IRI and their therapeutic potential.

## 2. Hepatic IRI

Hepatic IRI results from insufficient blood flow after ischemic storage/transportation, causing oxygen restriction following the restoration of blood flow, supporting the need for acute and fast oxygen and nutrient delivery during surgery [10,11]. It involves ischemia-mediated cellular damage and aggravated sterile inflammatory responses once blood flow is restored to ischemic hepatic tissue [9]. Hepatic IRI is classified as cold and warm IRI [9,12]. Cold IRI occurs post-donation during “cold storage” transportation of the organ, which slows down metabolism and preserves the organ and usually takes 6–10 h. Warm IRI occurs during liver transplantation surgery where there is an inhibition of blood flow to the organ for almost a half hour. During warm ischemia, the oxidative stress and immune cell response lead to hepatic cell injury with the cascade of inflammatory factors [9]. Hepatic IRI begins locally, but it can lead to liver failure and even multi-organ failure, especially when the inflammatory response becomes systemic [13,14].

An inflammatory response usually forms from tissue hypoxia triggering the production of reactive oxygen species (ROS) and the cascade of chemokines and cytokines leading to the activation of inflammatory genes [5]. Hepatic IRI is thought to be mediated by inflammatory response in conjunction with the activation of hypoxia-inducible factor (HIF) [4,14]. Then, active HIF transcription factors control the expression of multiples genes in response to oxygen concentrations in the downstream [4,15]. Kupffer cell activation is prominently expressed after warm ischemia, causing a release of proinflammatory cytokines [11]. Subsequently, the expression of transcription factor NF-κB leads to the formation of chemokines and further accumulation of immune cells [11]. Oxidative stress and ROS production in the liver result in the dysregulation of homeostasis and the necrosis of hepatocytes from neutrophil attacks [13,15].

The prevention and treatment of hepatic injury after transplantation is important to retrieve liver function and improve graft survival [4,16]. Liver-specific miRNA analysis has been shown to be promising for the detection of allograft dysfunction [2,17,18]. Manipulating miRNA levels could be used as a therapeutic approach for preventing inflammatory response and/or additional upregulation or downregulation of affected genes from IRI.

## 3. MicroRNA Biogenesis and Function

MicroRNAs (miRNAs) are a class of small non-coding RNAs composed of ~21 nucleotides [19]. The first miRNAs, lin-4 and let-7, were identified in 1993, leading to the discovery of thousands of miRNA species [20]. The function of miRNA was widely unknown until a further comprehension of its ability to impact gene expression was established. miRNAs have the capability to silence and alter the translation of all genes in the human genome and regulate transcriptional control [21,22]. A recent study shows the variability of miRNA expression levels from childhood to adulthood; therefore, miRNA is relevant for growth regulation and development processes [23]. In this regard, the role of extracellular miRNAs has been shown for cellular homeostasis by facilitating cell–cell communication [21]. With the discovery of additional tissue-specific miRNAs, other functions have been identified, leading to new possibilities for therapeutic agents and diagnostic tools [22].

The miRNA is synthesized endogenously inside the nucleus [22]. The primary miRNA (pri-miRNA) is transcribed from DNA and then cleaved by Drosha, an RNase III enzyme, which creates the pre-miRNA that is actively transported from the nucleus into the cytoplasm [22]. The direct pathway incorporates the Dicer enzyme to cleave the other end of the pre-miRNA, resulting in a mature double-stranded miRNA [22,24]. The Argonaute 2 protein (AGO 2) is then bound to allow the mature miRNA to produce an RNA-induced silencing complex (RISC), which interacts with miRNA response elements (MREs) [21,24,25]. The miRNA sequence usually guides the RISC complex to the complementary pair on the 3′ UTR of target mRNA. When bound to their target mRNA, gene expression will be inhibited through translational repression or mRNA degradation [22,24,26]. If the miRNA and MRE interaction is fully complementary, the mRNA is cleaved by the AGO-catalyzed degradation of the target mRNA sequence [27]. The ability for miRNA to have multiple binding sites on mRNA reveals that it has multiple pathways to target its translation and prompt degradation. In addition to its roles in gene expression regulation, miRNA has also been found to bind as ligands to the Toll-like receptor (TLR) family, resulting in an inflammatory response and potential tumor growth [28].

miRNA dysregulation is associated with cancer, neurodevelopmental disorders, organ transplantation, and additional diseases. Abnormal expression of miRNAs, whether the miRNAs are amplified or deleted, can be attributed to gene dysregulation and sustained aberrant signaling. The role of miRNA dysregulation was first identified as a hallmark of cancer then associated with neurodevelopmental disorders, organ transplantation, and additional diseases [28]. Some miRNAs are integral in the activation of cell proliferation leading to tumorigenesis, which is evident through the abnormal miRNA expression in tumors [21,28]. miRNA can function as an oncogene or tumor suppressor depending on the target genes.

## 4. miRNAs in Hepatic IRI

Various miRNAs have been identified in association with hepatic IRI that either exaggerate or ameliorate the hepatic IRI [2]. Altering targeted miRNA expression has great potential to reduce early graft dysfunction and improve patient outcome. Strategies to implement this approach have been studied using hepatic cell lines subjected to oxygen deprived conditions in vitro, as well as animal models after induction of hepatic IRI through warm ischemia in vivo [10]. By studying the mechanisms of specific miRNAs, the up- or downregulation during hepatic IRI reveals whether that miRNA can ameliorate or exaggerate the metabolism and functions of the liver. Alanine aminotransferase (ALT) and aspartate aminotransferase (AST) levels can be used to indicate when liver injury is present and improve diagnosis accuracy along with miRNA biomarkers [29]. The manipulation of miRNAs could have an influence on the inflammatory and oxidative stress pathways associated with hepatic IRI [2].

Chemically modified small RNAs can be used to mimic or antagonize miRNAs of interest. Agomirs can mimic miRNA to understand their function within an experiment. Antagomirs inhibit the function of the miRNA. Depending on the upregulation or downregulation of miRNAs that will attenuate the hepatic IRI, the corresponding agomir or antagomir can be applied as a therapeutic approach. Notable miRNAs that are upregulated during the pathogenesis of hepatic IRI include miR-122, miR-450-5b, miR-155, and miR-191. The upregulation of these miRNAs results in the inhibition of their target genes, resulting in inflammation and apoptosis. Additional miRNAs that will be further discussed in this review include miR-146a, miR-194, and miR-140-5p, which are downregulated during hepatic IRI.

### 4.1. miRNA-122

miR-122 is liver-specific and highly expressed, accounting for almost 70% of the miRNA present in the liver, but increases significantly during hepatic IRI [30,31,32]. Therefore, the alteration of the miR-122 level could be utilized as a biomarker to predict liver recovery after transplantation [33]. IRI causes the upregulation of miR-122 in correlation with higher alanine aminotransferase (ALT) and aspartate aminotransferase (AST) levels, which can serve as an indicator for liver injury [33]. miR-122 is regulated by the hypoxia-inducing factor-1α (HIF1α) transcription factor through the binding site within the miR-122 promoter [31].

In mice with HIF1α deletion, hepatocyte-specific miR122 initiation was prohibited [30]. With the complete deletion of miR-122 in mice, through isolating the dependence of HIF1α among miR-122 induction, studies have noted that prolyl hydroxylase domain (PHD1) is the target gene for miR-122 [30]. PHD1 is vital for maintaining protein stabilization through regulating HIF [30,31]. Through the ability to sense oxygen levels, PHD1 hydroxylates HIF1α except when there is lack of oxygen present; therefore, the tolerance for the body to handle hepatic ischemia is greatly reduced due to the repression of PHD1 levels when targeted by miR-122 [30]. Decreasing the levels of miR-122 by targeting HIF1α can be a promising therapeutic approach to promoting PHD1 expression and relief from hepatic IRI.

### 4.2. miRNA-450b-5p

miRNA-450b-5p suppresses Crystallin Alpha B (CRYAB), leading to increased inflammation stimulating hepatic IRI [34]. CRYAB has an anti-inflammatory impact on hepatic IRI by preventing IKKβ activation through reducing the canonical NF-κB pathway [34]. CRYAB can also relieve hepatic IRI through macrophage polarization targeting protein kinase B, which is inhibited by miRNA-450b-5p [34]. During hepatic IRI, miR-450-5p is upregulated in addition to IL-1β, tumor necrosis factor-α (TNF-α), and IL-6 [34]. Inhibiting miRNA-450b-5p could be used therapeutically to reduce severe inflammatory immune response and control hepatic IRI outcome in the clinic.

### 4.3. miRNA-155

miRNA-155 is upregulated by inflammatory mediators in association with innate and adaptive immune responses [18]. Mice with miR-155 deficiency had lower aminotransferase (ALT) levels [35]. One of the miR-155 targets is suppressors of cytokine signaling 1 (SOCS1). The downregulation of SOCS1 by miR-155 overexpression facilitates macrophage development and Th17 cell differentiation [35]. Liver macrophages trigger tissue inflammation and activate neutrophils specifically through macrophage phenotypes M1 and M2 [35]. M2 is the alternatively activated anti-inflammatory phenotype specifically used in tissue repair and homeostasis to enhance cell recovery that is upregulated when miR-155 is deficient [35,36]. miR-155 deletion also results in the suppression of IL-17 and inhibits the activation of Kupffer cells (KCs), resulting in a decrease in proinflammatory cytokines [37]. Overall, miR-155 deletion protects the mouse liver against IRI through the upregulation of SOCS1, resulting in less inflammation.

### 4.4. miRNA-191

Under ischemic/hypoxic conditions, miR-191 is upregulated, contributing to hepatic tissue damage and cell apoptosis [38]. miRNA-191 is also found to play a role in breast cancer through enhancing cell proliferation, migration, and chemoresistance [39]. When mice are subjected to hypoxia/reperfusion (H/R) stresses, miR-191 targets ZO-1-associated Y-box factor (ZONAB) [38]. miR-191 expression is mediated by hypoxia-inducible factor-1α (HIF-1α) at the promotor region [38]. ZONAB repression by miR-191 induces cell cycle arrest and apoptosis during hepatic IRI [38]. The miR-191 knockout mice showed less cell death and ischemic injury in comparison to the wild type [38]. The deletion of miR-191 appears to be a promising therapeutic approach to moderating liver tissue damage and cell death.

### 4.5. miRNA-370

During hepatic IRI, miRNA-370 is upregulated, causing inhibition of the transforming growth factor-β receptor II (TβRII) pathways and activation of the NF-κB pathway [40,41]. TβRII expression is a crucial receptor in the TGF-β signaling pathway through recruiting and phosphorylating the SMAD family of transcription factors [40]. Specifically through activation of SMAD 3, TNF-α and IL-1β expression are decreased while IL-10 is incre ased, inhibiting the activity of other inflammatory mediators to balance the immune response [42]. Hence, silencing miR-370 using antagomir-370 results in lower AST and ALT levels, indicating improvement for hepatic IRI [41].

### 4.6. miRNA-210

miR-210 was upregulated, leading to an increase in hepatocyte apoptosis during hepatic IRI [43]. miR-210 targets SMAD4 in the hypoxia pathway [43]. SMAD4 is suppressed when miR-210 is overexpressed. miR-210 knockout mice showed higher expression of SMAD4 and lower inflammatory markers and apoptosis [43]. Upon IRI, the miR-210 knockout mice had significantly lower ALT and AST levels in addition to lower TNF-α, IL-6, and IL-1β when compared to the wild-type mice [43]. Inhibition of miR-210 is a potential strategy for decreasing cell apoptosis and inflammatory responses during hepatic IRI.

### 4.7. miRNA-34

miR-34 is increased during hepatic IRI, which regulates Sirtuin 1 (SIRT1) expression along with p53 [44]. SIRT1 is an NAD+-dependent deacetylase that plays an important role by downregulating the inflammatory response and suppressing reactive oxygen species (ROS) [45]. While evaluating the miR-34/SIRT1 pathway, mice were pretreated with carbon monoxide (CO) inhalation as a preconditioning treatment to promote anti-inflammatory effects [44]. Mice treated with CO and subjected to an hour of warm ischemia followed by 6 h of reperfusion had lower ALT serum levels and decreased neutrophil accumulation in comparison to the control group [44]. The CO was able to inhibit miR-34a, resulting in increased SIRT1 expression, which represses apoptosis and inflammatory pathways [44]. In mice with SIRT1 knockout, pro-inflammatory cytokines were increased through NF-κB acetylation, proving the importance of SIRT1 expression [44]. The ability of CO to downregulate miR-34 is a promising therapeutic strategy through targeting the miR-34 pathway in the liver [44].

### 4.8. miRNA-497-5p

miR-497-5p expression is upregulated during hepatic IRI in mice, and its role is further evaluated in isolated Kupffer cells (KC) [46]. When miR-497-5p is inhibited in mice, hepatocyte apoptosis, following hepatic IRI, is decreased [46]. MED1 is suppressed when miR-497-5p is upregulated, resulting in decreased TIMP2 expression. In miR-497-5p knockout, MED1 overexpression results in the inhibition of NF-κB through TIMP2 expression, as well as lower TNF-α/IL-1β expressions [46]. When miR-497-5p is downregulated, hepatic IRI is ameliorated in association with MED1/TIMP-2 activation, showing potential treatment for alleviating liver injury.

### 4.9. miRNA-128-3p

miR-128-3p suppresses Rho family GTPase 3 (Rnd3), thus activating the NF-κB pathway and transcription factor p65 upon hepatic IRI [47]. miR-128-3p knockout mice had higher levels of Rnd3 and lower levels of TNF-α, IL-6, and AST in the serum [47]. When upregulated during hepatic IRI, miR-128-3p activates NF-κB signaling and downregulates SIRT1 expression, inducing oxidative stress [47,48]. Therefore, inhibiting miR-128-3p can ameliorate liver injury through the Rnd3/NF-κB axis.

### 4.10. miRNA-146a

One of the most important miRNAs involved in the inflammatory response is miRNA-146a [2,49]. During the development of IRI, miRNA-146a is downregulated, causing an increase in inflammatory cytokines. Among the key associated factors in hepatic IRI pathogenesis, the interleukin-1 receptor-associated kinase 1 (IRAK1) and tumor necrosis factor receptor-associated factor 6 (TRAF) are involved [50]. Specifically, miRNA-146a targets the Toll-like receptor 4 (TLR4) pathways by directly suppressing IRAK1 and TRAF6 [51]. The activation of TLR4 triggers a transmembrane signaling cascade, producing inflammatory cytokines including TNF-α and IL-6 [50]. It was shown that in mice, the overexpression of miR-146a protects the liver from hepatic injury [49,50]. miRNA-146a mimics could be used as a therapeutic agent to improve hepatic IRI by inhibiting the TLR signaling pathway, which leads to a decrease in the release of proinflammatory mediators.

Within the same miR-146 family, miR-146b is upregulated during hepatic IRI in contrast to the downregulation of miR-146a. miRNA profiles in porcine models of donation after brain death followed by circulatory death (DBCD) revealed an upregulation of miRNA-146b-5p [52]. Additional porcine models including donation after brain death (DBD) and donation after circulatory death (DCD) were evaluated in comparison to DBCD models and demonstrated that miR-7-1, miR-7-2, and miR-146b were significantly upregulated in the DBCD groups [52]. Further evaluated in 42 human samples, patients with high miR-146b expression also had EAD, revealing the potential use of miR-146b as a biomarker [52].

### 4.11. miR-194

miR-194 was down regulated in hepatic IRI, resulting in the upregulation of its target, pleckstrin homology-like domain family member 1 (PHLDA1) [53]. PHLDA1 activates TNF receptor-associated factor 6 (TRAF6), which exaggerates stress responses and inflammation during IRI through mitogen-activated protein (MAPK) initiation [53]. Impaired liver function was evident when PHLDA1 was overexpressed due to an increase in cytokines and chemokines [53]. miR-194 mimic and miR-194 antagomir were used to compare results to evaluate the mechanisms of the miR-194/PHLDA1 axis [53]. When miR-194 was overexpressed, ALT and AST levels were lower, along with the lower expression of TNF-α, IL-6, IL-1β, and CXCL-10 [53]. Targeting PHLDA1 through the overexpression of miR-194 has potential as a therapeutic agent to ameliorate liver damage.

### 4.12. miRNA-140-5p

To identify the role of miR-140-5p in hepatic IRI, overexpression and knockout mice models were used to compare the results [54]. The study revealed that miR-140-5p is downregulated during hepatic IRI, resulting in an increase in inflammatory markers and cell apoptosis [54]. Whenever miR-140-5p is overexpressed in cells, inflammatory cytokines and cellular apoptosis are reduced due to the inhibition of CAPN1 [54]. miR-140-5p negatively regulates CAPN1, which is activated during hepatic IRI, leading to the overactivation of signaling cascades and cellular damage [54]. AML12 cells were also subjected to conditions of hepatic IRI with hypoxia regeneration models to provide further understanding of miR-140-5p during liver injury [54]. Since miR-140-5p overexpression can reverse apoptosis and decreases ALT and AST levels, miR-140 supplementation is a potential way to achieve better outcomes in patients after liver transplantation.

### 4.13. Additional miRNAs

In addition to the miRNAs listed above, there are some miRNAs which were studied mainly in vitro using cell lines and a hypoxia/reoxygenation model. For example, miR-142-3p is downregulated upon hepatic IRI. miR-142-3p downregulation results in the upregulation of its targets including myristoylated alanine-rich C-kinase substrate (MARCKS) in vitro in HepG2 cells [55]. MARCKS upregulates NF-κB expression, causing the p38/JNK signal to exaggerate inflammation [55]. MiR-142-3p mimic was transfected into AML-12 and HepG2 cell lines after subjection to hypoxia reoxygenation conditions to evaluate the mRNA levels of MARCKS [55]. When miR-142-3p expression was increased, inflammation and apoptosis were decreased through the suppression of the MARCKS signal, thus proving to be a valuable treatment for hepatic IRI [55].

Another miRNA which was recently identified to have a role in hypoxia/reoxygenation in vitro is miR-297. Transformed human liver epithelial-2 (THLE-2) cells were used to investigate miR-297 function in hepatic IRI [56]. miR-297 suppresses SIRT3 and modulates oxidative stress pathways, resulting in NOD-like receptor pyrin domain containing 3 (NLRP3) activation and NF-κB phosphorylation [56]. A miR-297 antagomir displays a great potential to improve hepatic IRI by promoting SIRT3 expression and inactivating NLRP3.

Lastly, miR-9-5p was studied in liver sinusoidal endothelial cells (LSECs) in vitro. Cells were subjected to oxygen and glucose deprivation to investigate the role of miR-9-5p in hepatic IRI [57]. miR-9-5p was downregulated and CXC chemokine receptor-4 (CXCR4) was upregulated, leading to an inflammatory response and decreased cell survival rate [57]. When miR-9-5p was overexpressed due to miR-9-5p mimic transfection to LSECs, CXCR4 was greatly reduced and decreased TNF-α, IL-6, and IL-1β levels [57]. miR-9-5p overexpression might be a therapeutic approach for protecting LSECs from IRI.

## 5. Summary and Perspective

IRI is initiated through ischemia followed by an acute increase in oxygen during reperfusion. The development of IRI results in the dysregulation of biological pathways, causing impaired organ function and potential irreversible damage systemically. Due to the anaerobic conditions of IRI, the body undergoes oxidative stress, Ca^2+^ overload, inflammatory cascade, cytokine storm, and apoptosis. Hepatic IRI is an inevitable complication after liver transplantation that requires further research to identify prevention and treatment strategies to mitigate EAD. In this regard, miRNAs show promise as a diagnostic biomarker through the complexity of miRNA-mediated gene regulation by multifaceted pathways that either ameliorate or exacerbate pathological events. The effect of miRNA on the transcriptional level reveals the initiation of hepatic IRI pathogenesis and how miRNA regulation could be insightful for improving graft function.

**Figure 1:**
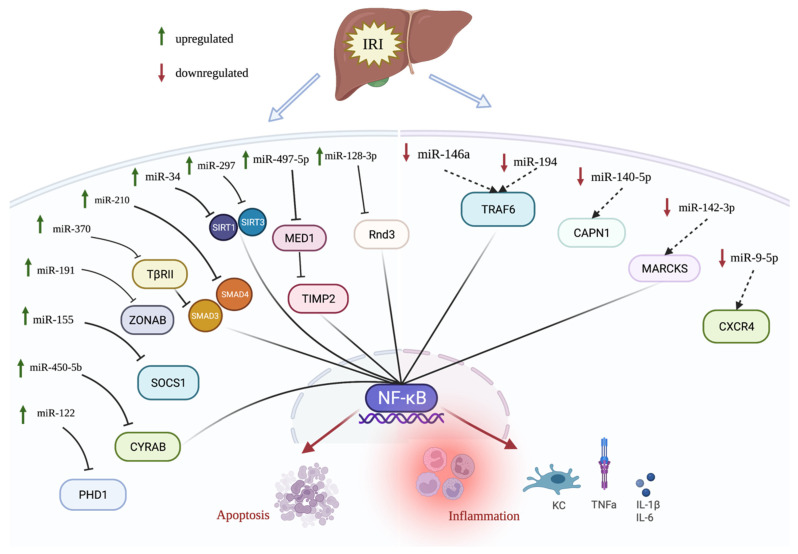
Differentially expressed miRNAs in hepatic IRI. miRNAs that are upregulated in hepatic IRI are indicated by green upward arrow, whereas miRNAs that are downregulated in hepatic IRI are indicated by downward red arrow. miRNAs that regulate NF-kB signaling are connected to NF-kB. For instance, miR-450-5b, miR-370, miR-34, miR-497-5p, and miR-128-3p are upregulated and inhibit their targets, causing an activation of NF-kB pathway during IRI. Reversely, miR-146a, miR-194, and miR-142-3p are downregulated in IRI, and their corresponding targets are upregulated, resulting in activation of NF-kB pathway.

A combination of various miRNA biomarkers based on their mRNA targets can reveal the main important pathways for the disease. Many of the miRNAs summarized in this review point to the prevalence of transcription regulator NF-kB during hepatic IRI (Figure 1). The upregulation of miR-450-5b, miR-370, miR-34, miR297, miR-497-5p, and miR-128-3p inhibits different target genes; however, they all activate NF-kB, resulting in increased inflammation and apoptosis. During hepatic IRI, the upregulation of miR-122, miR-155, miR-191, and miR-210 also increases the inflammatory response and cell apoptosis, but they do not directly target the NF-kB pathway. miR-146a, miR-194, and miR-142-3p are downregulated during IRI, causing an activation of their subsequent targets and ultimately activating NF-kB (Figure 1). mir-194 and miR-9-5p are also downregulated and result in inflammatory cytokine expression and cell death. When the downregulated miRNAs such as miR-146a, miR-194, and miR-142-3p are amplified in animal models, hepatic IRI is attenuated in comparison to the control. Hepatic IRI is also ameliorated by the inhibition of upregulated miRNAs such as miR-450-5b, miR-155, miR-3700, miR-210, and miR-34 (Table 1).

Evaluating miRNA’s role and function during hepatic IRI allows us to discover new therapeutic strategies to ameliorate liver injury. As summarized above, almost all miRNAs related to hepatic IRI have been typically studied using animal models, including mouse and porcine models or cell lines. There is a need to study human specimens collected either during liver resection or transplantation to identify which of the above miRNAs or novel miRNAs are changing and have consequences in human hepatic IRI. Most studies use smaller animal models, typically mouse models, which are only able to represent warm ischemia rather than the combination of cold and warm ischemia that is present in liver transplantation. The establishment of large clinical cohorts is necessary to investigate the presence or absence of specific miRNAs before and after transplantation.

In addition to tissue miRNAs, extracellular miRNAs can be evaluated as non-invasive biomarkers from biofluids including blood, serum, urine, bile, and perfusate [59]. However, many factors may affect the quality of RNA and downstream analyses. For example, plasma collection methods such as heparin vs. sodium-EDTA will affect the downstream analysis, as heparin inhibits the PCR-based methods. Processing and storage conditions will affect the RNA quality. The regulation and standardization of sample collection and storage, miRNA isolation and detection methods are necessary to identify effective miRNA biomarkers which are widely applicable and reproducible [60]. In addition to outside factors, patients’ conditions may also affect the levels of circulating miRNAs. For instance, dietary habits could impact the expression of miRNAs depending on their nutrition [61]. Tarallo et al. showed that healthy volunteers who are vegans or vegetarians had higher levels of miRNA-92a in their plasma compared to omnivore volunteers [62]. In parallel, Ferrero et al. observed a correlation between miRNA profile and natural compounds such as vitamin D or E intake in healthy volunteers [63]. Moreover, diets rich in polyunsaturated fatty acids and fat-soluble vitamins can lead to the expression of miRNAs which correlate with cardiovascular diseases and cancer development [61]. Thus, multiple parameters including sample collection, RNA isolation and detection methods, and personal lifestyles need to be considered when miRNAs are evaluated as biomarkers.

Various miRNAs have been studied as potential biomarker individually, but if we look at them collectively, there is a need to connect the common pathways to improve results. For instance, most of the miRNAs studied impact the inflammatory pathways through their specific targets; therefore, there is potential to further enhance patient outcomes through a combination of knockout and overexpression of miRNAs. Additional studies are needed to monitor the effects of the systemically applied designed miRNA agomirs and antagomirs to evaluate any unintentional, off-target effects in the genome. Evaluating the up- and downregulated miRNAs during human hepatic IRI reveals biological mechanisms which could be novel targets to improve patient outcome and quality of life post liver transplantation.

## Figures and Tables

**Table 1:** miRNAs and their function in hepatic IRI.

miRNAs	Change in IRI	Target	Effect on IRI	References
miR-122	Upregulated	PHD1	Exaggerates hepatic IRI when upregulated	[30,31,33,58]
miR-450b	Upregulated	CRYAB	Exaggerates hepatic IRI upon upregulation	[34]
miR-155	Upregulated	SOCS1	Exaggerates hepatic IRI when upregulated	[35,36,37]
miR-191	Upregulated	ZONAB	Exaggerates hepatic IRI when upregulated	[38,39]
miR-370	Upregulated	TβRII	Exaggerates hepatic IRI when upregulated	[40,41]
miR-210	Upregulated	SMAD4	Exaggerates hepatic IRI when upregulated	[43]
miR-34	Upregulated	SIRT1	Exaggerates hepatic IRI when upregulated	[44,45]
miR-297	Upregulated	SIRT3	Exaggerates hepatic IRI when upregulated	[56]
miR-497-5p	Upregulated	MED1/TIMP-2	Exaggerates hepatic IRI when upregulated	[46]
miR-128-3p	Upregulated	Rnd3	Exaggerates hepatic IRI when upregulated	[47]
miR-146a	Downregulated	TRAF6 and IRAK1	Prevents hepatic IRI when overexpressed	[49,50,51]
miR-194	Downregulated	PHLDA1	Prevents hepatic IRI when overexpressed	[53]
miR-140-5p	Downregulated	CAPN1	Prevents hepatic IRI when overexpressed	[54]
miR-142-3p	Downregulated	MARCKS	Prevents hepatic IRI when overexpressed	[55]
miR-9-5p	Downregulated	CXCR4	Prevents hepatic IRI when overexpressed	[57]

## Data Availability

Not applicable.
